# Application of Allometric Scaling and Translational Modeling to Predict Human Pharmacokinetics of mRNA-Encoded Antibodies

**DOI:** 10.1021/acs.molpharmaceut.5c01615

**Published:** 2026-04-26

**Authors:** Tam N. T. Nguyen, Philip K.-Y. Chang, Paul Panorchan, Uğur Şahin, Özlem Türeci, Shu-Pei Wu

**Affiliations:** † 461225 BioNTech US Inc., 40 Erie Street, Suite 110, Cambridge, Massachusetts 02139, United States; ‡ 435117 BioNTech SE, An der Goldgrube 12, Mainz 55131, Germany

**Keywords:** allometric scaling, mechanistic model, first-in-human prediction, mRNA therapeutics, RiboMab

## Abstract

Messenger RNA (mRNA)-lipid nanoparticle (mRNA-LNP) platforms enable the in vivo expression of almost any therapeutic protein, offering unprecedented flexibility for clinical translation. However, for these rapidly deployable therapies, predicting the first-in-human (FIH) dose remains a key challenge. We developed an allometric scaling framework for human pharmacokinetic (PK) prediction of antibodies expressed from intravenously administered mRNA-LNPs, leveraging preclinical and clinical data for BNT141 (encoding RiboMab01, a full IgG1) and BNT142 (encoding RiboMab02.1, a bispecific Fab-scFv-based T-cell engager). The dose-normalized Cmax (DCmax) and dose-normalized AUC (DAUC) of translated antibodies across multiple species could be described by the allometric approach, with the estimated exponents ranging from −1.29 to −1.42 for BNT141 and BNT142. We also determined generalized single-species allometric scaling exponents of −1.26 from mice and −0.75 from nonhuman primate (NHP), respectively, that enabled the human predictions of translated antibodies exposure approximately 4-fold (mice) and within 2-fold (NHP). A mechanistic cross-species translational model was further developed that integrated mRNA-specific elimination and translation efficiency parameters to characterize the exposure of the translated antibodies from mRNA-based therapeutics. The translational model could predict the human PK exposure of translated antibodies within 1.33-fold for BNT141 and BNT142 via single-species scaling from NHP parameters as well as capturing the concentration-time profiles with reasonably high fidelity. Validation with publicly available mRNA-1944 (encoding CHKV-24, full IgG1) data confirmed both the allometric scaling and translational modeling methods’ robustness when scaling from NHP data. Taken together, these results support a generalized cross-species PK relationship that is independent of the molecular characteristics of the translated antibodies or antibody-like protein products. This integrated scaling and modeling framework offers a generalizable solution for accelerating FIH dose selection of mRNA-encoded therapeutic antibodies and is adaptable to other recombinant proteins of interest.

## Graphical abstract

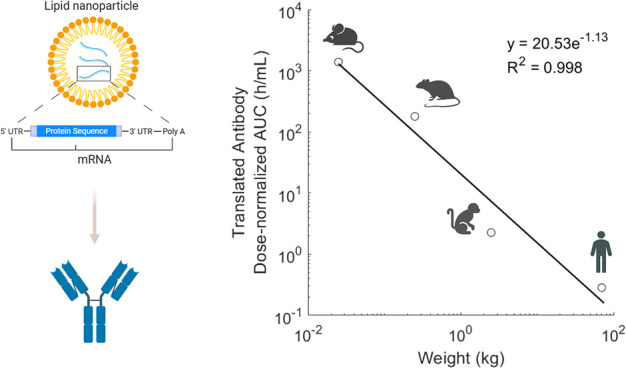

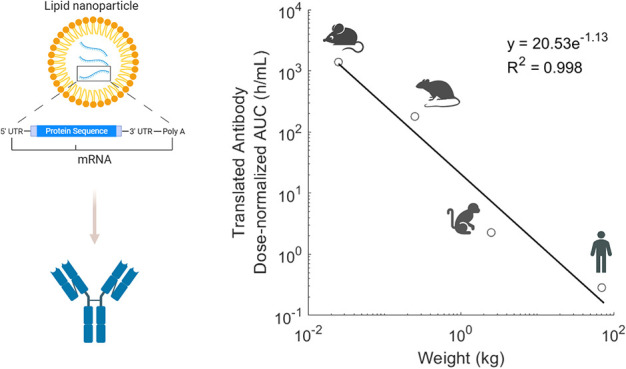

## Introduction

Messenger RNA (mRNA)-lipid nanoparticle (LNP) platforms represent a transformative approach for the in vivo expression of therapeutic proteins. In this system, a nucleoside-modified mRNA encoding the protein of interest is encapsulated within an LNP and administered intravenously. Following systemic delivery, the LNPs are primarily taken up by hepatocytes, where the mRNA is translated into the encoded protein and subsequently secreted into the circulation if the protein contains a secretion signal.^1,2^ This modular technology enables the expression of virtually any recombinant protein (including complex molecules such as monoclonal antibodies) using the same manufacturing process, independent of the encoded sequence.

This flexibility is particularly attractive for therapeutic antibodies, which we term *RiboMabs* when expressed in vivo from mRNA-LNPs. Compared to traditional antibody production, the mRNA-LNP approach offers rapid development timelines, lower manufacturing costs, and the ability to evaluate multiple antibody candidates in the clinic without the need to establish separate cell lines or production processes for each molecule.^1^ However, for these benefits to be fully realized, the determination of the first-in-human (FIH) dose must be both rapid and reliable, ensuring that dose selection does not become a bottleneck in clinical translation.

Allometric scaling is commonly used to predict the FIH dose in various small- and large-molecule therapeutic modalities. For small molecule drugs, allometric scaling is well-defined and widely accepted as industry standard to predict human dose.^3–5^ Allometric scaling has also been successfully developed for extrapolating preclinical data from different animal species to humans for monoclonal antibodies (mAbs) despite the complex pharmacokinetic (PK) behavior of mAbs that may involve target-mediated nonlinear elimination and recycling by the neonatal Fc receptor.^6–9^ Recently, an allometric scaling model has also shown promising capability for human dose prediction in adeno-associated viral vector gene therapies.^10^ Despite the widespread use of allometric scaling in predicting human dose, its application has been limited in mRNA-based therapies, with only a few reports on using preclinical data to predict the human dose.^11–13^ Notably, the human dose prediction using allometric scaling in mRNA-based therapeutics has not yet been validated by PK data in humans.

In this work, we focus on two investigational mRNA-LNP therapeutics that encode *RiboMabs* and are being clinically evaluated in cancer patients. BNT141 encodes RiboMab01, whose sequence is identical to zolbetuximab, which is a full IgG1 antibody that targets the cancer-associated gastric-lineage marker CLDN18.2.^2^ BNT142 encodes RiboMab02.1, a bispecific-fragment antibody and T cell engager that targets CD3 and the tumor-associated antigen CLDN6.^14^ Both mRNA-LNPs employ a nucleoside-modified noninflammatory mRNA backbone optimized for stability and translational efficiency, formulated with the same LNP technology for efficient hepatic delivery.^2,14^ Following intravenous administration, both candidates achieve systemic antibody exposure via liver-mediated secretion. In addition to these internal datasets, we incorporated published PK data for mRNA-1944, which is generated with a different LNP formulation from BNT141/142 and encodes CHKV-24, an anti-Chikungunya virus monoclonal antibody, providing an external benchmark for testing the generalizability of our approach.^15,16^

The PK profiles of translated antibodies from BNT141 and BNT142 in preclinical species^2,14^ and in human were used to developed a platform allometric scaling approach as well as a translational modeling approach, validated with the external data from mRNA-1944, to predict FIH dose for mRNA-encoded antibodies and antibody-like proteins. The allometric scaling and mechanistic modeling results suggest a generalized relationship for PK across different species independent of translated monoclonal antibodies or other antibody-like protein products. This model provides the foundation that is broadly applicable as a generalized allometric scaling and modeling approach for predicting human PK exposure using data from NHP regardless of the translated antibody, mRNA sequence, and LNP encapsulation method.

## Materials and Methods

### BNT141 Data

BNT141 encodes RiboMab01, an antibody whose sequence is identical to IMAB362 (zolbetuximab). IMAB362/zolbetuximab has a molecular weight of approximately 147 kDa and is a full IgG1 antibody that targets the cancer-associated gastric-lineage marker CLDN18.2.^17^ The DNA sequence for RiboMab01 was human codon-optimized and based on the known IMAB362 antibody heavy and light chain sequence.^2^ Each transcribed RNA strand contains common structural elements optimized for stability and translational efficiency: CC413 as 5′-cap, AGA-dEarI-hAg as 5′-untranslated region (UTR), FI element as 3′-UTR, and a poly­[A] tail measuring 100 adenosines with a linker at position 30 [A30L70].^2^ The transcribed RNA was encapsulated in LNP (ionizable cationic lipid, a polyethylene glycol (PEG) lipid, phospholipid, and cholesterol) to generate BNT141.^2^ Typically, the LNP sizes were approximately 70 nm and the polydispersity index <0.1.^2^

The preclinical PK profiles of the translated RiboMab01 from BNT141 in mouse, rat and monkey were obtained as previously reported.^2^ In rodents and NHP, repeated dosing of BNT141 resulted in sustained exposure of RiboMab01 without a decrease in protein-producing capacity and without significant safety findings.

BNT141-01 (NCT04683939) is a phase I/II, open-label, multisite, dose escalation and expansion trial to examine safety, efficacy, and PK/pharmacodynamics (PD) of BNT141 in pretreated patients with advanced unresectable or metastatic CLDN18.2-positive solid tumors that failed at least first line standard-of-care (SOC) therapy prior to enrollment. In the BNT141-01 study, serum concentrations of BNT141-encoded RiboMab01 were analyzed from serum samples collected from study participants at predose, end of infusion, 3, 6, 24, 48, 72, 168, 336, and 504 h following BNT141 IV administration. All participants provided written informed consent. PK parameters including Cmax, AUCs (AUC_0‑inf_, AUC_0‑t_, AUC_0‑last_, and/or AUC_0‑τ_), and half-life were derived using noncompartmental analysis (NCA) performed in Phoenix WinNonlin version 8.3.4 (Certara) and were summarized by dose level using descriptive statistics (*n*, arithmetic mean, s.d., CV, median, geometric mean, geometric CV, minimum and maximum). Exposure parameters (Cmax and AUCs) were also normalized based on the total administered dose to obtain dose-normalized Cmax and AUCs (DCmax and DAUCs). DAUC_0‑inf_ was used for evaluation in this manuscript. BNT141-01 PK data show that repeated dosing of BNT141 in human resulted in sustained translation of RiboMab01.

### BNT142 Data

BNT142 encodes a bispecific T-cell engager antibody designated as RiboMab02.1 (molecular weight of approximately 100 kDa), which binds to the tumor-associated antigen (TAA) Claudin 6 (CLDN6) and the T-cell antigen cluster of differentiation 3 (CD3).^14^ BNT142 design contains a hAg-Kozak 5′UTR, anti-CLDN6 VH and VL encoding sequences derived from mAb206-SUBS, also referred to as IMAB206-C46S, and no tag sequences.^14^ To prevent unfolding and aggregation, the anti-CLDN6 scFv moieties were stabilized by additional disulfide bridges.^14^ Full RNA sequences of BNT142 and amino acid sequences of RiboMab02.1 polypeptide chains are available.^18^ The RNA was encapsulated by an LNP formulation consisting of an ionizable cationic lipid, a PEG, lipid, phospholipid, and cholesterol, into BNT142 RNA-LNPs with sizes of 60 to 120 nm.^14^

The preclinical PK profiles of the translated RiboMab02.1 from BNT142 in mouse and monkey have previously been reported.^14^ Repeated dosing of BNT142 in mice resulted in sustained exposure of RiboMab02.1 without significant safety findings. Single dosing of BNT142 in NHP resulted in prolonged RiboMab02.1 exposure and was well tolerated.

BNT142-01 (NCT05262530) is a phase I/II, open-label, multicenter trial to evaluate the safety, tolerability, PK/PD and preliminary efficacy of BNT142 treatment in pretreated patients with CLDN6+ advanced solid tumors that failed at least first line SOC therapy prior to enrollment. In the BNT142-01 study, serum concentrations of BNT142-encoded RiboMab02.1 were analyzed from serum samples collected from study participants at predose, end of infusion, 3, 6, 10 (optional), 24, 48, and 168 h following BNT142 administration. BNT142-01 PK data show that repeated dosing of BNT142 in human also resulted in sustained translation of RiboMab02.1. All participants provided written informed consent. PK parameters including Cmax, AUCs, and half-life were derived using NCA performed in Phoenix WinNonlin version 8.3.4 (Certara) and were summarized by dose level using descriptive statistics (*n*, arithmetic mean, s.d., CV, median, geometric mean, geometric CV, minimum and maximum). Exposure parameters (Cmax and AUCs) were also normalized based on the total administered dose to obtain dose-normalized Cmax and AUCs (DCmax and DAUCs). DAUC_0‑inf_ was used for evaluation in this manuscript.

### mRNA-1944 Data

The preclinical and healthy volunteers’ clinical data (NCT03829384) of the translated CHKV-24 antibody from mRNA-1944 were extracted from the published materials.^15,16^ The preclinical concentration-time profile data of CHKV-24 is only available in NHP. PK parameters of NHP data including Cmax and AUCs were derived using NCA performed in Phoenix WinNonlin version 8.3.5 (Certara) and were summarized by dose level using descriptive statistics (*n*, arithmetic mean, s.d., CV, median, geometric mean, geometric CV, minimum and maximum). Exposure parameters (Cmax and AUCs) were also normalized based on the total administered dose to obtain DCmax and DAUCs. DAUC_0‑inf_ was used for evaluation in this manuscript.

### Simple Allometry of Pharmacokinetic Parameters

Regression analyses of the DCmax and DAUC were performed according to the power function described in eq 1:

$$P=P_{0}x+W^{\alpha }$$ 1

where *P* is the parameter of interest, *P*_0_ is the coefficient, α is allometric exponent and *W* is the body weight in kilograms. For preclinical species, the typical bodyweight values were used: 0.025 kg for mouse, 0.25 kg for rat,^19^ and 2.5 kg for nonhuman primate (NHP). For human body weights, a typical human body weight of 70 kg was used.

### Evaluation of Generalized Exponents for Human Prediction via Allometry

The predicted values of DCmax and DAUC for the translated antibodies of BNT141 and BNT142 were calculated using allometric scaling, using exponents from −1.3 to −1 for mice, and −0.9 to −0.6 for NHP. For each exponent, the fold-difference between the predicted values (pred) and the observed data (obs) was calculated. The prediction errors were calculated as the fold-difference of the predicted over the observed values in human. The geometric means of all prediction errors from BNT141 and BNT142 for DCmax and DAUC were used as the metric to evaluate the overall prediction performance and select the generalized exponent for each species. A predicted ratio within 2-fold is generally considered as acceptable for model performance.^20^

### Modeling and Allometric Scaling Method

Simple allometry was performed in MATLAB R2024b Update 5 (24.2.0.2863752) 64-bit. A custom translational mechanistic model, which included mRNA and translated antibody kinetics, was developed to capture the cross-species concentration-time profiles of the translated antibodies. The translational modeling work was performed in NONMEM version 7.4 (Icon Inc., PA) using a nonlinear mixed-effects modeling approach. The translational model parameters were estimated using first-order conditional estimation with interaction. Datasets and graphics were constructed using R version 4.1.3.

## Results

### Simple Allometric Scaling

The interspecies (rodents, NHP, and humans) allometric scaling results for the exposure of translated RiboMab01 from BNT141 and RiboMab02.1 from BNT142 are shown in Figure 1. For BNT141, an inverse relationship with respect to the species weight was found for both DCmax and DAUC: the multispecies allometric exponent was −1.29 for DCmax and −1.13 for DAUC (Figure 1A). For BNT142, a similar inverse relationship was also observed between DCmax and DAUC versus weight across multiple species (rodents, NHP, and humans) in translated RiboMab02.1 (Figure 1B) with allometric exponent of −1.42 and −1.38, respectively.

**Figure 1:**
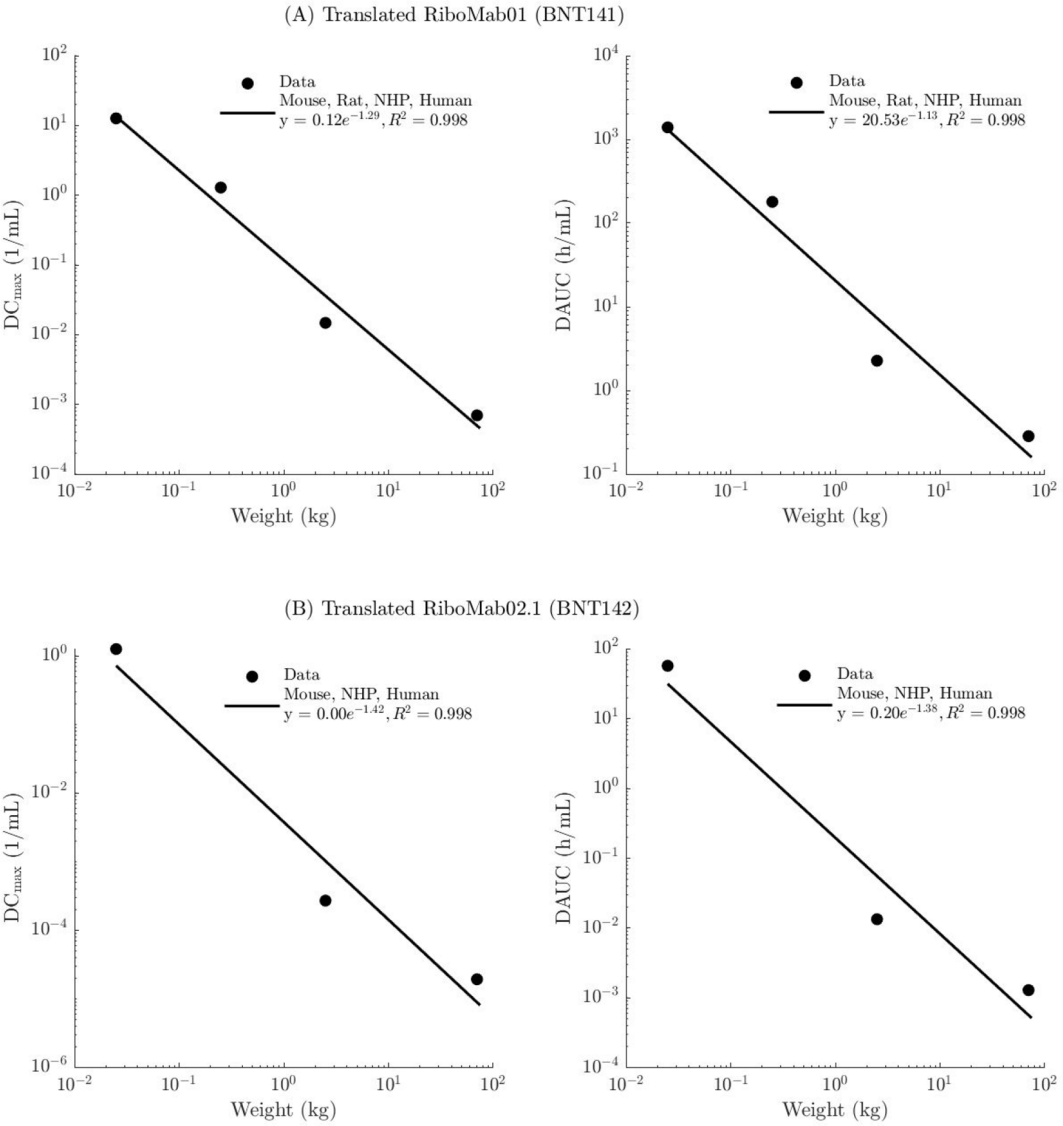
Allometric scaling of interspecies PK parameters for the translated antibodies: (A) RiboMab01 (BNT141) and (B) RiboMab02.1 (BNT142).

To determine the appropriate allometric exponents that could be used for a generalized cross-species-based approach, a range of exponents was explored to predict human PK parameters from mouse or NHP. The mean prediction errors of the exponents are shown in Figure 2. Based on this result, the generalized exponents were determined to be −1.26 and −0.75 for allometric scaling from mouse or NHP, respectively, for DCmax and DAUC. The single-species-based generalized exponents could predict both DCmax and DAUC from NHP data for BNT141 and BNT142 within 2-fold of the observed value for all PK parameters, while the human exposure predictions from single-species generalized exponent derived from mouse could be up to 4.44-fold for select PK parameter (Table 1). Further validation with external mRNA-1944 data shows that the single-species exponent from NHP to human derived from BNT141 and BNT142 data could also predict the DCmax and DAUC of translated CHKV-24 within approximately 2-fold of the observed value (Table 1).

**Figure 2:**
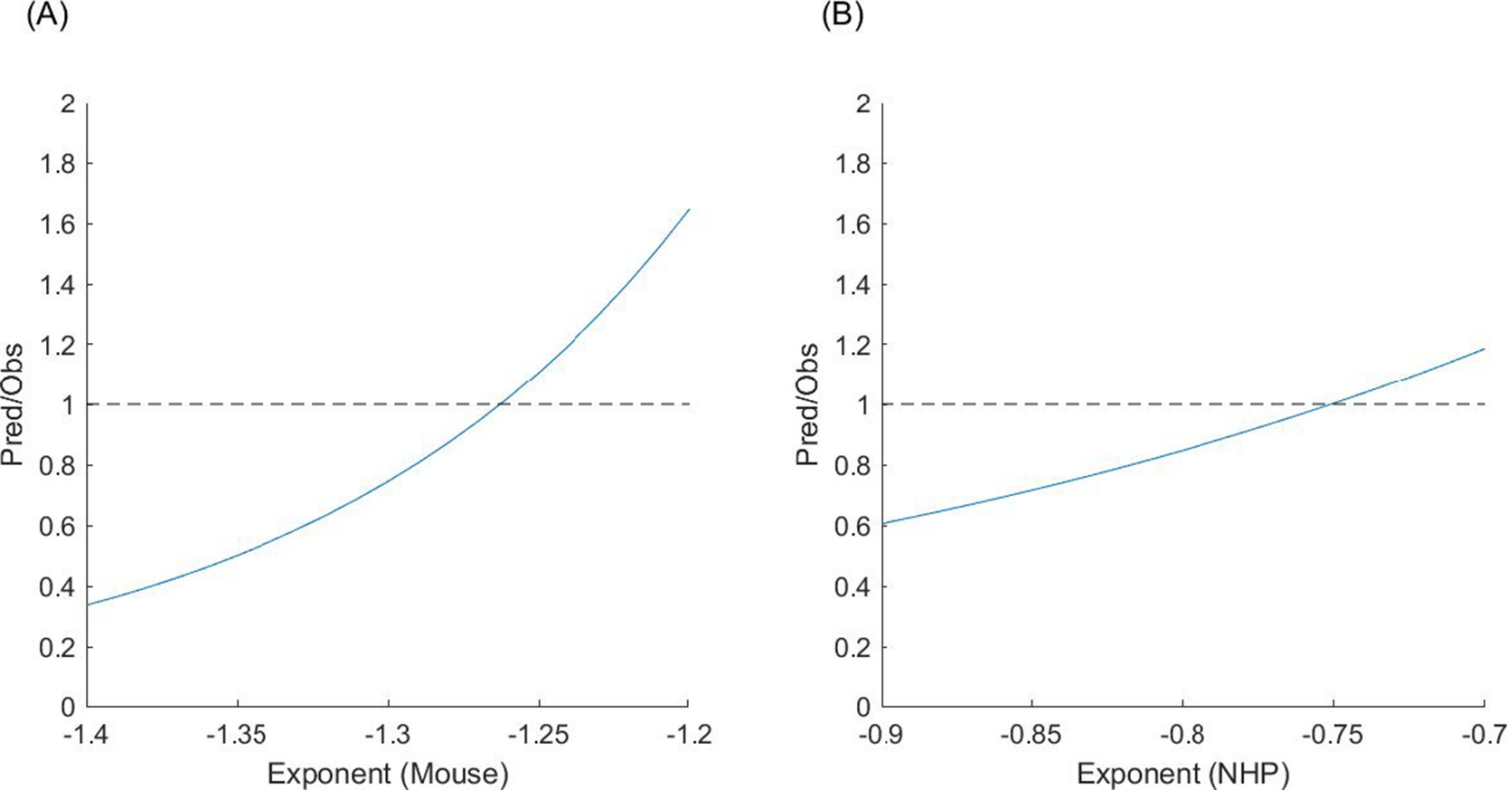
Mean prediction errors of translated antibodies PK (combined statistics for both BNT141 and BNT142 using all DAUC and DCmax values). (A) Mean prediction error from mouse. (B) Mean prediction error from NHP.

**Table 1:** Comparison between Observed PK Parameters and Allometric Scaling Predictions

compound	parameter	observed Value	predicted human value from mouse (prediction error as Pred/Obs)^a^	predicted human value from NHP (prediction error as Pred/Obs)^a^
BNT141	DCmax (1/mL)	6.96 × 10^–4^	5.72 × 10^–4^	1.15 × 10^–3^
			(↓ 1.22)	(↑ 1.65)
	DAUC (h/mL)	0.28	0.063	0.18
			(↓ 4.44)	(↓ 1.56)
BNT142	DCmax (1/mL)	1.93 × 10^–5^	5.70 × 10^–5^	2.23 × 10^–5^
			(↑ 2.95)	(↑ 1.16)
	DAUC (h/mL)	1.29 × 10^–3^	2.6 × 10^–3^	1.10 × 10^–3^
			(↑ 2.03)	(↓ 1.17)
mRNA-1944	DCmax (1/mL)	3.02 × 10^–4^		5.23 × 10^–4^
				(↑ 1.73)
	DAUC (h/mL)	0.42		0.19
				(↓ 2.21)

a The arrows indicate (↓) underestimation or (↑) overestimation and the numeric values within the paratheses reflect the fold differences of the predicted values relative to the observed data.

### Interspecies Translational Mechanistic Modeling

To better understand the dynamics of the translated antibodies from mRNA across species and improve human prediction, a translational model was proposed to dissect the antibody disposition and antibody translation kinetics. The translational mechanistic model was developed to include the mRNA-LNP elimination kinetics, translation of mRNA-LNP into antibody, and antibody disposition kinetics to capture the general process of mRNA-LNP pharmacokinetics and mRNA translation into antibodies. mRNA-LNP was assumed to be eliminated through the processes of enzymatic elimination, liver uptake, etc., which was characterized by a first-order elimination rate (*k*_elimination, mRNA_). The translation of mRNA-LNP component into antibody (*k*_translate_) was described through a first-order reaction and the mRNA-LNP was not consumed as the antibody was synthesized. The translated antibody PK was described by a typical 2-compartment disposition model with first-order elimination from the central compartment. The antibodies disposition parameters were the translated antibodies clearance *CL*_Ab_, central volume of distribution *V*_1_, peripheral volume of distribution *V*_2_, and intercompartmental clearance *Q*_Ab_. The model does not take into account the potential limitation of liver production and/or organ stress limiting translational activities over time. The model scheme is demonstrated in Figure 3 and model equations are shown in Table 2. Simple allometry scaling function (eq 1) was applied on model parameters (Table 1) to describe the changes across species with respect to body weight (*W*), where α_*i*_ was the allometric exponent with respect to model parameter *i*, i.e., α_*V*_1__ was the allometric exponent with respect to parameter *V*_1_ and so on. Random effects were modeled as exponential terms representing log-normal distributions of the parameters. Residual variability was accounted for by a proportional error model for the translated antibodies concentration.

**Figure 3:**
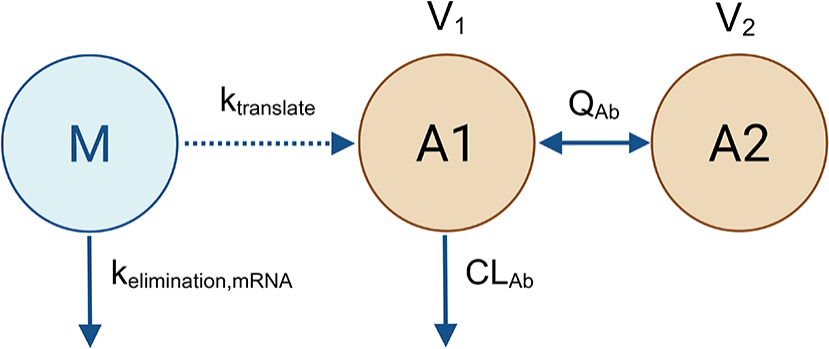
Translational model structure.

**Table 2:** Model Equations

model equation	corresponding state
$\frac{dM}{dt}=-k_{\mathrm{elimination},\mathrm{mRNA}}\cdot W^{\alpha_{k_{\mathrm{elimination},\mathrm{mRNA}}}}\cdot M$ (eq 2)	mRNA-LNP amount (*M*)
$\frac{dA_{1}}{dt}=k_{\mathrm{translate}}\cdot W^{\alpha_{k_{\mathrm{translate}}}}\cdot M-{\mathrm{CL}}_{\mathrm{Ab}}\cdot W^{\alpha_{{\mathrm{CL}}_{\mathrm{Ab}}}}\cdot \frac{A_{1}}{V_{1}\cdot W^{\alpha_{V_{1}}}}-Q_{\mathrm{Ab}}\cdot W^{\alpha_{Q_{\mathrm{Ab}}}}\cdot \left(\frac{A_{1}}{V_{1}\cdot W^{\alpha_{V_{1}}}}-\frac{A_{2}}{V_{2}\cdot W^{\alpha_{V_{2}}}}\right)$ (eq 3)	translated antibodies in central compartment (*A*_1_)
$\frac{dA_{2}}{dt}=Q_{\mathrm{Ab}}\cdot W^{\alpha_{Q_{\mathrm{Ab}}}}\cdot \left(\frac{A_{1}}{V_{1}\cdot W^{\alpha_{V_{1}}}}-\frac{A_{2}}{V_{2}\cdot W^{\alpha_{V_{2}}}}\right)$ (eq 4)	translated antibodies in peripheral compartment (*A*_2_)

The model was calibrated to the concentration-time profiles of translated RiboMab01 after dosing BNT141 in mouse, rat, NHP and human, and RiboMab02.1 after dosing BNT142 in mouse, NHP, and human. The estimated parameters and scaling exponents are included in Table 3. For BNT142, only one elimination phase was observed, thus *V*_2_ and *Q*_Ab_ were not included in the case of RiboMab02.1. The scaling exponents for the volumes of distribution (*V*_1_, *V*_2_) in both cases were assumed to follow the typical value and fixed at 1,^6^ while the rest of the exponents were estimated. For BNT141, the values of *V*_1_ and *V*_2_ were fixed to the parameter estimates of the reference protein IMAB362/zolbetuximab using data from mouse, rat and human (Table S1 and Figure S1).^2,21^ For BNT142 the value of *V*_1_ was fixed to the average central volume of distribution parameter estimate of select bispecific antibodies^22–27^ (Table S2).

**Table 3:** Translational Model Parameters

		RiboMab01 (BNT141)		RiboMab02.1 (BNT142)	
parameter (unit)	parameter description	parameter estimate (% RSE)	scaling exponent estimate (% RSE)	parameter estimate (% RSE)	scaling exponent estimate (% RSE)
*k*_elimination, mRNA_ (1/h/kg)	mRNA-LNP clearance	0.041 (13%)	–0.14 (42%)	0.0282 (2%)	–0.042 (13%)
*k*_translate_ (1/h/kg)	antibodies synthesis rate from LNP/mRNA	0.27 (12%)	–0.43 (8%)	0.043 (19%)	–0.59 (8%)
*CL*_Ab_ (mL/h/kg)	antibody clearance	0.28 (10%)	0.79 (5%)	7.68 (21%)	0.80 (6%)
*V*_1_ (mL/kg)	antibody central volume of distribution	40.1 (fixed)	1 (fixed)^6^	58.4 (fixed)	1 (fixed)^6^
*V*_2_ (mL/kg)	antibody peripheral volume of distribution	45.6 (fixed)	1 (fixed)^6^		
*Q*_Ab_ (mL/h/kg)	antibody intercompartmental clearance	1.02 (17%)	1.03 (21%)		

The final model parameter estimates are shown in Table 3. The estimated exponent for *CL*_Ab_, ∼0.80 in both cases, was comparable to published exponents for antibodies.^6^ The estimated exponents for the translation rate were −0.43 and −0.59 for RiboMab01 and RiboMab02.1, respectively, indicating an inverse relationship between translational efficacy and weight. The scaling exponent for the elimination rate of mRNA was estimated at −0.14 and −0.04 for RiboMab01 and RiboMab02.1, respectively. The overlays of predicted PK profiles with observed PK profiles are shown in Figures 4 and 5, indicating that the model adequately captured the data. The model was able to capture PK of translated antibody from single and multiple dosing of BNT141 and BNT142 in rodents, NHP and human.

**Figure 4:**
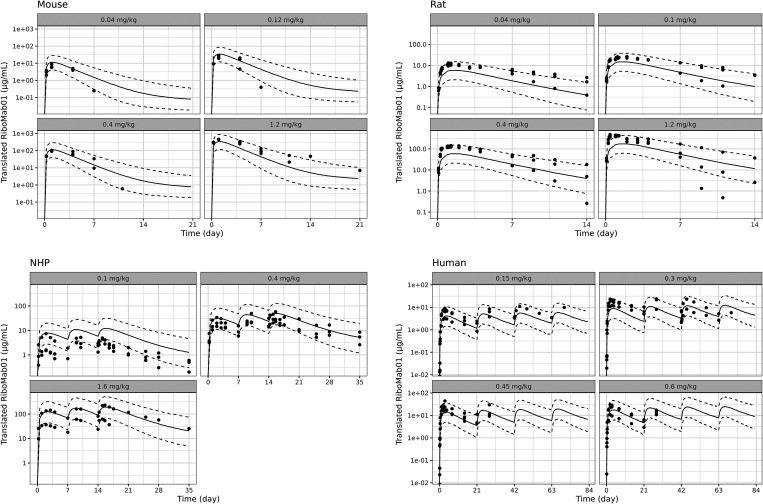
Translational model simulation vs data of translated RiboMab01 from BNT141. Solid line indicates the prediction median. Dotted line indicates the 90th percentile and 10th percentile of model prediction. Dots indicate data points measured in the studies.

**Figure 5:**
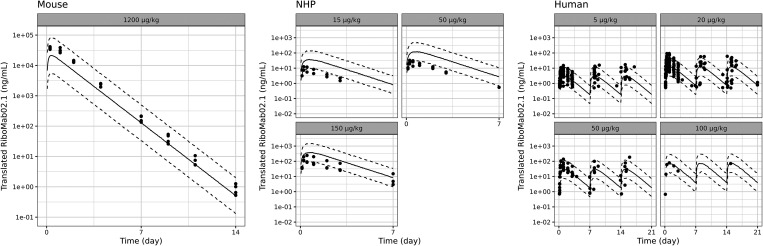
Translational model simulation vs data of translated RiboMab02.1 from BNT142. Solid line indicates the prediction median. Dotted line indicates the 90th percentile and 10th percentile of model prediction. Dots indicate data points measured in the studies.

We applied single-species allometric scaling to the translational model parameters obtained from mouse and NHP data (Tables S3 and S4) to obtain and evaluate the human concentration-time profile predictions. For both BNT141 and BNT142, an exponent of −0.10 was applied for *k*_elimination, mRNA_ (average of estimated exponents in Table 3); an exponent of 0.80 was applied for *CL* (average of estimated exponents in Table 3); an exponent of 1 was applied for volumes *V*_1_ and *V*_2_; and an exponent of 0.65 was applied for *Q*.^6,28–30^ For singles-species prediction from mouse, the exponent −0.51 was applied for the antibody translational rate parameter *k*_translate_ (average of estimated exponents in Table 3), and no adjustment was made to the translation rate (exponent of 0) when performing single-species prediction from NHP following the allometric scaling results. The single-species scaling approaches could reasonably predict the concentration-time profiles in human for both translated RiboMab01 and RiboMab02.1 following BNT141 and BNT142 repeated dosing (Figure 6). In addition, the model predicted values for DCmax and DAUC were within 3.59-fold compared to the observed data when using mouse data and 1.33-fold when using NHP data (Table 4).

**Figure 6:**
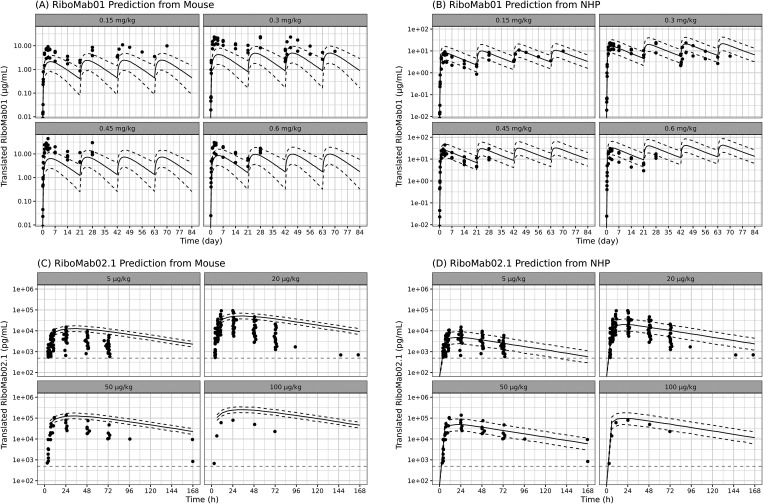
Single-species model predictions vs observed data. (A, B) mRNA-LNP translated RiboMab01 prediction from mouse and NHP. (C, D) mRNA-LNP translated RiboMab02.1 prediction from mouse and NHP. Solid lines indicate the median model prediction. Black dotted lines are 90 and 10% model prediction. Gray dashed line indicates the lower limit of quantification.

**Table 4:** Model Prediction vs. Observed Data of Translated Antibodies Exposure

			human prediction value and error (Pred/Obs)^a^	
compound	parameter	observed human	using mouse data	using NHP data
BNT141	DAUC (h/mL)	0.28	0.078	0.33
			(↓3.59)	(↑1.17)
	DCmax (1/mL)	6.96 × 10^–4^	2.13 × 10^–4^	8.00 × 10^–4^
			(↓3.27)	(↑1.15)
BNT42	DAUC (h/mL)	0.0013	0.0035	0.0012
			(↑2.71)	(↓1.08)
	DCmax (1/mL)	1.93 × 10^–5^	3.63 × 10^–5^	1.45 × 10^–5^
			(↓1.88)	(↓1.33)
mRNA-1944	DAUC (h/mL)	0.42		0.28
				(↓1.35)
	DCmax (1/mL)	3.02 × 10^–4^		2.23 × 10^–4^
				(↓1.52)

a The arrows indicate (↓) underestimation or (↑) overestimation and the numeric values within the paratheses reflect the fold differences of the predicted values relative to the observed data.

To further validate the human prediction methodology, the model was applied to predict the human PK profile of translated CHKV-24 following mRNA-1944 dosing, using PK data from NHP.^15,16^ The model parameters in NHP in mRNA-1944 are included in Table S5. The predicted profile and reported clinical data are shown in Figure 7. The modeling approach could adequately predict the profile for the first 56 days as well as the general trend over the course of 420 days for the translated CHKV-24. The model could predict the PK data of CHKV-24 over single and multiple dosing regimen upon mRNA-1944 administration. The predicted exposure DCmax and DAUC in humans were within 1.52-fold of the reported clinical data (Table 4). This result reinforced the single-species translational modeling approach to predict human dose using NHP data.

**Figure 7:**
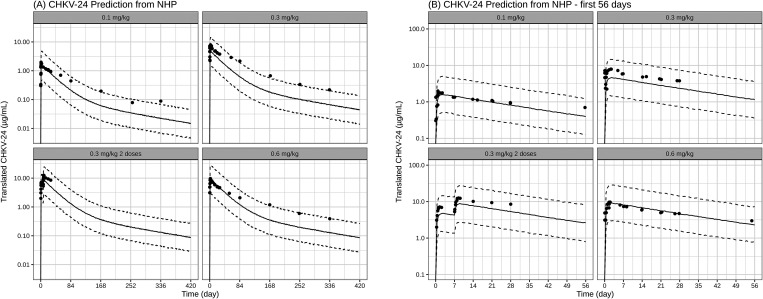
Single-species model predictions vs observed. (A) mRNA-LNP translated CHKV-24 prediction from NHP and (B) mRNA-LNP translated CHKV-24 prediction from NHP for the first 56 days. Solid lines indicate the median model prediction. Black dotted lines are 90 and 10% model prediction.

## Discussion

Our study sought to establish a framework for translating preclinical PK data of mRNA-LNP-encoded antibodies to humans. Applying allometric scaling on multispecies DCmax and DAUC for BNT141 and BNT142, we found that both PK parameters scale with negative exponents (inversely) with body weight across species. These negative exponents indicate that exposure per unit dose decreases in larger species. The similarity of the estimates across two very different antibodies with potential pharmacological differences—RiboMab01 being a full IgG1 and RiboMab02.1 being a bispecific antibody fragment—suggests the allometric scaling methodology can be generalized and applied to mRNA-encoded antibody therapeutics despite their potential differences in structure and design.

As single-species allometric scaling methods can provide a useful and practical tool for human prediction, we identified generalized exponents for scaling from individual preclinical species. Using BNT141 and BNT142 data, an exponent of −1.26 enabled prediction of human exposure from mouse data while an exponent of −0.75 was sufficient when extrapolating from NHPs. The difference in exponents for mouse and NHP indicates that there is considerable difference in the translational efficiency between mouse and human compared to NHP and human, given that the clearance of the translated antibodies can be allometrically scaled like recombinant antibodies^9^ and the exponent of single-species allometry can be viewed as a combination of mRNA translational process plus antibodies elimination. Predictions from mice carried a wider error margin (up to 4-fold), which could be attributed to the uncertainty due to limited data, the larger body-weight gap between mice and humans that magnifies even small errors in the exponent, and the potentially larger differences in the translational efficiency of mRNA-LNP between mice and humans compared to NHPs and humans, as demonstrated in a hepatocytes study.^31^ Despite the higher prediction error, rodent models remain valuable for early drug-discovery programs due to their accessibility and low cost. In contrast, NHP-based scaling with the −0.75 exponent consistently predicted human exposures with an approximately 2-fold error margin for BNT141, BNT142 as well as the external mRNA-1944 data, arguing for NHPs as the preferred species when higher precision is required. The generalized NHP-based exponent for scaling mRNA-encoded antibodies is similar to the commonly adopted exponent for scaling monoclonal antibody clearance;^6^ this similarity suggests that the translational rate is comparable between NHPs and humans, and that the exposure of other mRNA-encoded proteins may also be scaled similarly to their recombinant counterparts. Validation with the external dataset demonstrated that the single-species NHP-based scaling relationship may be generalized beyond our internal *RiboMab* platform.

To distinguish the contributions of intravenously administered mRNA PK and antibody disposition, we developed a translational model, calibrated on BNT141 and BNT142 data, that describes the elimination of mRNA-LNP, the mRNA-to-antibody translation and the disposition of translated antibody. The translational modeling results show that the exponents for scaling translated protein disposition parameters are similar to recombinant protein PK. An exponent of −0.51 was estimated across species for the antibody translation rate per mRNA drug unit *k*_translate_ (Table 3), which represented the net kinetic rate of the translation-related processes and can be understood as the translational efficiency. The negative exponent on *k*_translate_ in multispecies characterization suggests that translational efficiency decreases in larger species and remains a significant factor driving cross-species differences in exposure to mRNA-encoded antibodies along with antibody clearance. This trend mirrors previous findings that translational efficiency or metabolic power decreases with increasing body weight: Zou et al. derived an allometric relationship between gene efficiency factor with respect to species weight with exponent −0.25 for adeno-associated viral vector therapies;^10^ the metabolic rate of cells in vivo in mammalian species has been reported to decrease with increasing body weight with an allometric exponent of −0.25;^32^ and the mitochondrial transcript reported to scale with body weight with an allometric exponent between −0.28 and −0.4.^33^ Our findings, along with the aforementioned studies, suggest that translational efficiency correlates with interspecies differences in cellular translation machinery. The single-species translational model can reproduce the full concentration-time profiles with good fidelity for BNT141, BNT142 and mRNA-1944 using NHP-based scaling. We suggest that the framework may be adapted to describe and predict human pharmacokinetics from NHP data for other intravenously administered mRNA-encoded therapeutic proteins translated from the liver, beyond antibodies or antibody-like protein products. This adaptation can be accomplished by scaling the mRNA-LNP elimination parameter using the exponent from our study without scaling the translation parameter, in addition to scaling the antibody or protein disposition parameters as appropriate.

To our knowledge, this work is the first study to validate the human predictions with clinical PK data and take into account preclinical species differences to human across multiple molecules. In previous studies that performed FIH prediction, standard single-species allometric scaling exponents were used on either mRNA kinetics or translated antibody PK without consideration of species differences;^11,13^ only mRNA PK differences in preclinical species but not protein PK differences were considered;^12^ and translated antibody PK predictions were not compared with clinical data in these studies. Our work expands in three important aspects: (1) we derived exponents specifically from mRNA-encoded antibody data across multiple species; (2) we incorporated an mRNA-specific translation efficiency parameter with species-dependent scaling; and (3) we validated single-species scaling from both mice and NHPs using human clinical data. These advances provide a more biologically grounded basis for dose selection than other approaches that assume constant translation efficiency across species.

In preclinical settings, a dose of 10 μg BNT141 administered via IV injection in mice could produce a comparable Cmax to an IV dose of 80 μg IMAB362 (reference protein) in mice.^2^ For BNT142, IV administration of 1.4 mg/kg BNT142 in mice achieved a similar Cmax of RiboMab02.1 compared to a 2.5 mg/kg IV dose of the reference protein. In a xenograft mouse study, a dose of 0.1 μg BNT142 in mice was able to achieve comparable antitumor effects to 100 μg of the reference protein.^14^ Whether mRNA-based therapeutics will be able to achieve comparable clinical exposure to injectable proteins remains to be investigated and studied.

Several limitations warrant discussion. First, our dataset is limited to molecules with liver-targeting LNP; whether the same exponents apply to mRNA constructs targeting other tissues remains to be seen. Second, the estimated exponents represent average behavior and may not capture outliers such as antibodies with target-mediated disposition or saturable elimination.^6^ Third, our model focuses on single-dose PK and does not account for immunogenicity or adaptive immune responses that could alter translation efficiency or clearance upon repeat dosing.^34^ Nevertheless, the close agreement between model predictions and observed human data across distinct antibodies, despite potential differences in translation-driving mRNA-backbones across all three molecules as well as in LNP formulations of BN141/142 and mRNA-1944, suggests that the identified scaling relationships adequately describe the PK characteristic of mRNA-encoded antibodies.

In conclusion, our work demonstrated a generalized allometric scaling relationship for mRNA-encoded antibodies. We presented a platform approach for determining the human PK exposure from NHP PK data independent of the translated antibody structure and LNP formulation, and predicting human PK using mouse PK data, which can expedite the drug development timeline and increase the chance of clinical success. The framework of this approach can potentially be adapted to predict the human exposure of other mRNA-encoded proteins simply by taking into account the recombinant protein disposition PK. Broader validation with additional molecules and delivery systems will help refine the exponents and confirm the utility of this platform in accelerating the development of mRNA-based therapeutics.

## Supplementary Material



## Data Availability

Supporting data are available upon reasonable requests and are subject to review.

## Notes

Additional information supporting the mechanistic modeling results, including model parameter estimates in mouse and NHP (PDF)
