# Supplementary material for: Application of Allometric Scaling and Translational Modeling to Predict Human Pharmacokinetics of mRNA-Encoded Antibodies
Source: Mol Pharm. 2026 Apr 26;23(6):3254–64. doi: 10.1021/acs.molpharmaceut.5c01615 (PMC13231417; doi:10.1021/acs.molpharmaceut.5c01615)
Supplement: Supplementary file 1 [file mp5c01615_si_001.pdf]

Supporting information

Application of allometric scaling and translational modeling to predict human pharmacokinetics of mRNA-encoded antibodies

Tam N.T. Nguyen[1*], Philip K.-Y. Chang[1], Paul Panorchan[1], Uğur Şahin[2], Özlem Türeci[2], Shu-Pei Wu[1*]

[1]BioNTech US Inc., 40 Erie Street, Suite 110, Cambridge, MA 02139, USA

[2]BioNTech SE, An der Goldgrube 12, 55131 Mainz, Germany

*Corresponding author:

Tam N.T. Nguyen / +1-617-337-4701 / tam.nguyen@biontech.us / BioNTech US Inc., 40 Erie Street, Suite 110, Cambridge, MA 02139, USA

Shu-Pei Wu / +1-617-337-4701 / shu-pei.wu@biontech.us / BioNTech US Inc., 40 Erie Street, Suite 110, Cambridge, MA 02139, USA

This document includes additional information supporting the mechanistic modeling results, including model parameter estimates in mouse and NHP.

Table S1. Model parameters of recombinant anti-CLDN18.2 RiboMab01.

Parameter	*Unit*	*Mouse*	*Rat*	*Human*[a]
CL_{Ab}	mL/h/kg	0.22 (5%)	0.278 (9%)	0.182 (11%)
Q_{Ab}	mL/h/kg	2.18 (19%)	1.49 (9%)	0.29 (11%)
V_1	mL/kg	44.2 (10%)	27.1 (6%)	52.1 (4%)
V_2	mL/kg	41.8 (11%)	39.1 (11%)	56 (28%)

[a]Human data were extracted from phase 1 dose-escalation study of IMAB362[1].

Table S2. Volume of distribution of select approved bispecific antibodies.

**Compound**	**Description**	$\boldsymbol{V_1}$ **(L)**	$\boldsymbol{V_1}$ **(mL/kg)**[a]
Tarlatamab[3]	DLL3 x CD3	3.4[3]	48.57
Cibisatamab	CEA x CD3	3.87[4]	55.29
Mosunetuzumab	CD20 x CD3	5.49[5]	78.43
Teclistamab	BCMA × CD3	4.13[6]	59.00
Glofitamab	CD20 × CD3	3.33[7]	47.57
Talquetamab	BCMA x CD3	4.32[8]	61.43

[a]Assuming a typical human bodyweight of 70kg.

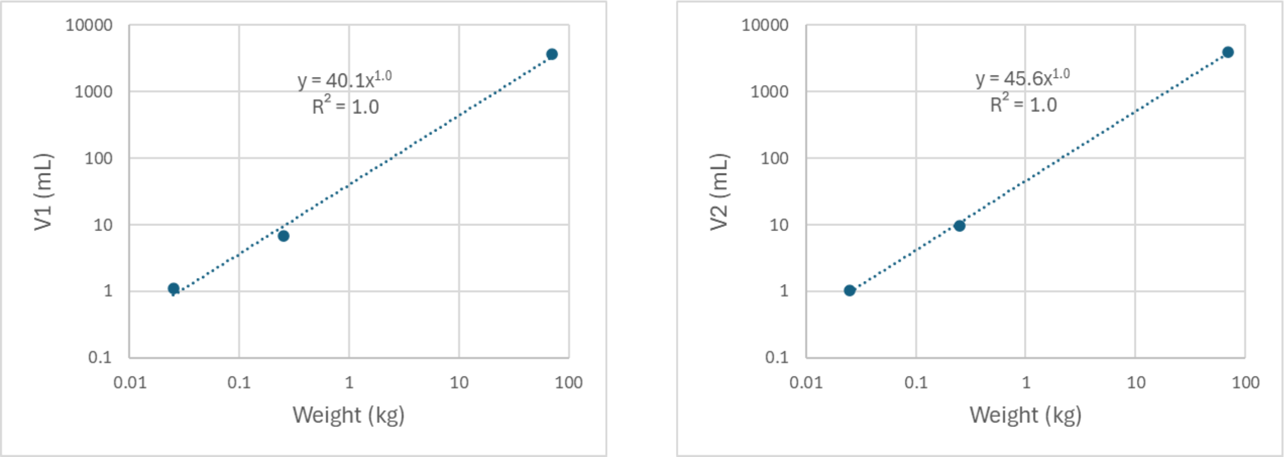

Figure S1. Allometric scaling of volumes of distribution parameters for the recombinant protein of BNT141.

Table S3. Estimated model parameters of translated RiboMab01 from BNT141 dosing.

Parameter	*Unit*	*Mouse*	*NHP*
$k_{\mathrm{elimination,mRNA}}$	1/h/kg	1.36 (13%)	0.020 (19%)
$k_{\mathrm{Translate}}$	1/h/kg	36.9 (11%)	0.045 (18%)
CL_{Ab}	mL/h/kg	1.06 (20%)	0.24 (12%)
Q_{Ab}	mL/h/kg	0.072 (22%)	0.078 (33%)
V_1	mL/kg	44.2 (Fixed)[a]	44.3 (Fixed)[b]
V_2	mL/kg	41.8 (Fixed)[a]	40.0 (Fixed)[b]

[a]Value estimated from recombinant RiboMab01 PK (Table S1).
[b]Volumes of distribution were assumed to be the common median values for monoclonal antibodies with a typical 70 kg human bodyweight[2]

Table S4. Estimated model parameters of translated RiboMab02.1 from BNT142 dosing.

Parameter	*Unit*	*Mouse*	*NHP*
$k_{\mathrm{elimination,mRNA}}$	1/h/kg	1.34 (1%)	0.0086 (7%)
$k_{\mathrm{Translate}}$	1/h/kg	27 (9%)	0.0037 (12%)
CL_{Ab}	mL/h/kg	14.6 (10%)	13.0 (12%)
V_1	mL/kg	58.4 (Fixed)[a]	58.4 (Fixed)[a]

[a]Volumes of distribution were assumed to be the average of select approved bispecific antibodies with a typical 70 kg human bodyweight (Table S2)

Table S5. Estimated model parameters of translated CHKV-24 from mRNA-1944 dosing.

Parameter	*Unit*	*NHP*
$k_{\text{elimination,mRNA}}$	1/h/kg	0.037 (15%)
$k_{\text{Translate}}$	1/h/kg	0.020 (31%)
CL_{Ab}	mL/h/kg	0.079 (21%)
Q_{Ab}	mL/h/kg	0.035 (37%)
V_1	mL/kg	44.3 (Fixed)[a]
V_2	mL/kg	40.0 (Fixed)[a]

[a]Volumes of distribution were assumed to be the common median values for monoclonal antibodies[2].

References

(1) Sahin, U.; Schuler, M.; Richly, H.; Bauer, S.; Krilova, A.; Dechow, T.; Jerling, M.; Utsch, M.; Rohde, C.; Dhaene, K.; Huber, C.; Türeci, Ö. A Phase I Dose-Escalation Study of IMAB362 (Zolbetuximab) in Patients with Advanced Gastric and Gastro-Oesophageal Junction Cancer. *European Journal of Cancer* **2018**, *100*, 17–26. https://doi.org/10.1016/j.ejca.2018.05.007.

(2) Ryman, J. T.; Meibohm, B. Pharmacokinetics of Monoclonal Antibodies. *CPT Pharmacom & Syst Pharma* **2017**, *6* (9), 576–588. https://doi.org/10.1002/psp4.12224.

(3) Kong, S.; Minocha, M.; Chen, P.-W.; Martinez, P.; Anderson, E. S.; Parkes, A.; Houk, B. E.; Lin, C.-W. Population Pharmacokinetics of Tarlatamab, a Half-Life Extended DLL3-Directed Bispecific T-Cell Engager in Patients with Previously Treated Small Cell Lung Cancer. *Clin Pharmacokinet* **2025**, *64* (5), 729–741. https://doi.org/10.1007/s40262-025-01499-z.

(4) Sanchez, J.; Pierrillas, P. B.; Frey, N.; Lotz, G. P.; Jönsson, S.; Friberg, L. E.; Frances, N. A Model-Based Approach to Evaluate Anti-Drug Antibody Impact on Drug Exposure With Biologics: A Case Example With the CD3 T-Cell Bispecific Cibisatamab. *CPT Pharmacom & Syst Pharma* **2025**, *14* (6), 1065–1076. https://doi.org/10.1002/psp4.70019.

(5) Bender, B.; Li, C.; Marchand, M.; Turner, D. C.; Li, F.; Vadhavkar, S.; Wang, B.; Deng, R.; Lu, J.; Jin, J.; Li, C.; Yin, S.; Wei, M.; Chanu, P. Population Pharmacokinetics and CD20 Binding Dynamics for Mosunetuzumab in Relapsed/Refractory B-cell non-Hodgkin Lymphoma. *Clinical Translational Sci* **2024**, *17* (6), e13825. https://doi.org/10.1111/cts.13825.

(6) Miao, X.; Wu, L. S.; Lin, S. X. W.; Xu, Y.; Chen, Y.; Iwaki, Y.; Kobos, R.; Stephenson, T.; Kemmerer, K.; Uhlar, C. M.; Banerjee, A.; Goldberg, J. D.; Trancucci, D.; Apte, A.; Verona, R.; Pei, L.; Desai, R.; Hickey, K.; Su, Y.; Ouellet, D.; Samtani, M. N.; Guo, Y.; Garfall, A. L.; Krishnan, A.; Usmani, S. Z.; Zhou, H.; Girgis, S. Population Pharmacokinetics and Exposure-Response with Teclistamab in Patients With Relapsed/Refractory Multiple Myeloma: Results From MajesTEC-1. *Target Oncol* **2023**, *18* (5), 667–684. https://doi.org/10.1007/s11523-023-00989-z.

(7) Shirley, M. Glofitamab: First Approval. *Drugs* **2023**, *83* (10), 935–941. https://doi.org/10.1007/s40265-023-01894-5.
(8) U.S. Food and Drug Administration, Center for Drug Evaluation and Research. *Multidiscipline Review: Application No. 761342*; 2023. https://www.accessdata.fda.gov/drugsatfda_docs/nda/2023/761342Orig1s000MultidisciplineR.pdf (accessed 2024-05-17).
